# Multilevel HierCC typing scheme for global genomic epidemiology and antimicrobial resistance surveillance of *Campylobacter jejuni*

**DOI:** 10.1128/aac.00310-26

**Published:** 2026-07-28

**Authors:** Ruochen Wu, Sandeep Kaur, Michael Payne, Li Zhang, Ruiting Lan

**Affiliations:** 1 School of Biotechnology and Biomolecular Sciences, University of New South Wales 7800, Sydney, NSW, Australia; Universita degli Studi di Roma La Sapienza, Rome, Italy

**Keywords:** *Campylobacter jejuni*, multilevel HierCC typing, antimicrobial resistance, genomic surveillance

## Abstract

*Campylobacter jejuni* is the most common bacterial cause of human gastroenteritis around the world. A stable, scalable, and standardized typing scheme is essential for epidemiological and antimicrobial resistance (AMR) surveillance for prevention and control of *C. jejuni* infections. We curated, assembled, and quality-filtered a *C. jejuni* global whole-genome sequencing (WGS) data set of 63,012 publicly available Illumina short-read genomes. A *C. jejuni* global cgMLST scheme with 1,161 core loci was developed using a representative subset of 2,587 genomes. We compared the HierCC clustering of the cgMLST profiles at every allelic distance, and identified nine epidemiologically meaningful levels to form a novel multilevel HierCC typing (MHT) scheme. AMR prediction was performed for the global data set using AbritAMR and integrated with the MHT scheme. The multilevel design provides flexible typing resolution for both short- and long-term epidemiological investigations and allows natural and genetically discrete clusters to be described consistently at both global and local scales. We showcased the utility of the scheme with the identification of country- and continent-specific clusters at HC264 to HC19 levels and differentiation of farm-animal-specific (specialist) from human infection (generalist) clusters at HC264 to HC55 levels. In the U.S. dataset from 2016 to 2022, a total of 276 antibiotic-resistant clusters were identified, with 22 resistant clusters increasing in frequency in recent years. An increased prevalence of *C. jejuni* carrying *50S_L22* mutations in the USA was observed from 2016 to 2022, suggesting continuing selection pressure from macrolide use in farm animals.

## INTRODUCTION

*Campylobacter jejuni* is a motile, microaerophilic, Gram-negative bacterium with global prevalence (1). It is a zoonotic pathogen commonly recovered in most livestock and animal food products (2, 3). Human transmission predominately occurs through the consumption of undercooked or contaminated poultry products or unpasteurized dairy (3, 4). It is the leading bacterial cause of gastroenteritis in the world, impacting 96 million people annually by the estimation of the World Health Organization (WHO) (5–7). *C. jejuni*-induced gastroenteritis has also been associated with long-term illness, such as dysbiosis, irritable bowel syndrome (IBS), and Guillain-Barré syndrome (GBS) (8, 9).

Antibiotics are rarely prescribed for *C. jejuni* infections as most cases are mild and self-limiting (4). However, antibiotics are warranted for patients with severe gastrointestinal infections, with persistent fever, bloody diarrhea for 7 days or more, significant volume loss, and having eight or more bowel movements per day (4, 10, 11). In such cases, the drug of choice is a macrolide, specifically erythromycin or azithromycin, as recommended by the American Academy of Pediatrics and the Infectious Diseases Society of America (4, 7, 10–12).

In 2017, the European Union (EU) issued an action plan to reinforce the need for comprehensive data collection, monitoring, and surveillance of AMR (13). The increasing emergence of antimicrobial resistance (AMR) and multidrug resistance (MDR) in *C. jejuni* has been extensively documented, which has become a major public health concern in both developed and developing countries in recent years (7, 14–17). Antibiotic stewardship in the United Kingdom (UK) has been associated with some reduction in antibiotic resistance in farm animals, but the regulatory oversight in the United States of America (USA) has led to antibiotic overuse (18–22). The impact of inadequate AMR surveillance and regulation on public health is likely to be more severe in developing countries (23).

Rapid advancement in next-generation sequencing (NGS) technologies has made whole-genome sequence (WGS) data more affordable and accessible than ever before (24). Currently, the USA and the UK are the only two countries (25–27) that have provided large amounts of WGS data in the public domain from their national pathogen surveillance programs utilizing NGS technology. Molecular typing techniques such as multilocus sequence typing (MLST) and core genome MLST (cgMLST) have contributed significantly to short- and long-term *C. jejuni* epidemiological studies (28, 29). MLST has played a pivotal role in elucidating the genetic diversities of *C. jejuni* in both evolutionary and ecological studies (30, 31). The *C. jejuni* global population consisted of both globally and locally distributed MLST sequence types (STs), with ST-21, ST-45, and ST-50 being the most frequently sampled STs around the world (32, 33). The subsequent introduction of the Oxford cgMLST scheme for *C. jejuni* and *C. coli* has provided high-resolution typing necessary for investigating local *C. jejuni* epidemiology (29, 34–36). However, both MLST and cgMLST schemes only offer fixed resolution, with no scalability. MLST lacks the resolution needed to differentiate closely related isolates, while the resolution of cgMLST could be too high for surveillance over longer time frames (37, 38)

HierCC is a method that clusters cgMLST allelic profiles into multilevel hierarchical clusters (39). Since 2018, HierCC has been implemented in EnteroBase to support a variety of functions across multiple hierarchical levels, from differentiating species/subspecies at low-resolution levels to identifying individual transmission chains at high-resolution levels (40). The resolution flexibility of HierCC enables utility in both long-term and short-term epidemiological analyses, as demonstrated in *Salmonella*, *Escherichia/Shigella*, *Clostridioides*, *Vibrio*, *Yersinia*, and *Mycobacterium* in the EnteroBase (41). However, a HierCC scheme for *C. jejuni* is currently absent, and there is no simple means to determine which levels are most suitable for describing bacterial population structure and epidemiology. In this study, we develop a cgMLST specifically for *C. jejuni* and identify nine HierCC levels to create a stable multilevel HierCC typing (MHT) scheme that allows for scalable resolution with epidemiologically relevant divisions of the *C. jejuni* population. We defined country- or continent-specific clusters, farm-animal-specific clusters, and resistant clusters to medically important antibiotics for the global *C. jejuni* population. Importantly, we demonstrated the utility of the MHT scheme for AMR surveillance when incorporated with national WGS surveillance data from the UK and the USA.

## MATERIALS AND METHODS

### Curation of Illumina genomic data set

Publicly available *C. jejuni* raw Illumina paired-end reads (*n* = 68,657) and metadata were downloaded and processed from the European Nucleotide Archive (ENA https://www.ebi.ac.uk/ena, June 2023) (42). The processing and assembly of raw reads were performed using the shovill (v1.1.0) and strategic k-mer extension for scrupulous assemblies (SKESA) (43–48). The assembled genomes were quality-filtered based on thresholds in Methods S1. *Campylobacter* species confirmation of the assembled genomes was performed using the CampyGStyper (49). The final *C. jejuni* global data set consisted of 63,102 genomes that passed the quality control filtering.

### Development of the *C. jejuni* global cgMLST scheme

We developed a *C. jejuni*-specific cgMLST using a smaller data set termed the representative data set (*n* = 2,587), which aimed to capture the global genetic variation of *C. jejuni*. All assembled genomes in the global data set were assigned the pubMLST *Campylobacter* seven-gene MLST STs using the MLST package (https://github.com/tseemann/mlst) (50). The representative data set was populated by randomly selecting one genome from each unique MLST STs (*n* = 2,587) in the global data set (*n* = 63,102). The cgMLST scheme was constructed using the representative data set and the Comprehensive and Highly Efficient Workflow BSR-Based Allele Calling Algorithm (chewBBACA v3.3.2) with default parameters. The core loci were defined based on the typeability in the representative data set (*n* = 2,587). Only loci present in 95% of genomes in the representative data set were retained in the scheme (detailed description in Methods S2). We named the final scheme the UNSW *C. jejuni* cgMLST scheme (available at https://github.com/LanLab/C.jejuni-multilevel-HierCC-typing-scheme).

### Implementation of HierCC in *C. jejuni* global data set

HierCC v1.24 was used to cluster 63,102 *C. jejuni* genomes based on the cgMLST allelic profiles at default setting (39). The HierCC package HCCeval was modified in Python to output the allelic profile distance matrix to reduce overall run time of large data set. At each HierCC level, the silhouette score of the HierCC clusters and the distance matrix was calculated using the scikit-learn package silhouette_score (51). The silhouette score was calculated based on allelic profile distances between isolates to measure the similarity of isolates within and between the HierCC clusters, with values ranging from −1 to +1. The higher the silhouette score, the better the separation of the clusters. The adjusted Rand index (ARI) between the clustering of isolates at each HierCC level and the division of isolates in the seven gene MLST were calculated using the scikit-learn package adjusted_rand_score (51). The ARI measures the stability of clustering between two HierCC levels.

### Antimicrobial resistance annotation

The genetic determinants conferring antibiotic resistance of the assembled genomes were identified using AbritAMR (version 1.0.14) at default setting (52). The resistance determinant genes or mutations responsible for phenotypic resistance were only included if the coverage was ≥90% and nucleotide identities ≥90%. Phenotypic interpretation of the 15 antibiotic classes and antibiotic combinations was predicted based on the mechanisms of the resistance determinants. The validity of phenotype resistance prediction by AbritAMR was tested using WGS of 3,181 isolates with known phenotype AMR profiles from the NCBI Pathogen Detection database in June 2023 (https://www.ncbi.nlm.nih.gov/pathogens). For macrolide resistance, only strains carrying *erm*(B) and mutations in the 23S rRNA gene are classified as resistant. Although mutations in *50S_L22* are also detected and annotated using AbritAMR, they are analyzed separately as indicators of macrolide selection pressure, as they do not confer resistance by themselves.

### Resistance score and its statistical comparison between different data subsets

The calculation of resistance score (RS) and statistical comparison of resistance rates of antibiotic classes or combinations were performed using a Python (version 3.9.6) script from AMR analysis (53). The RS value for each genome was calculated by converting the susceptibility to each of the 15 antibiotic classes into Boolean values, where susceptible was registered as 0 and resistant was registered as 1. The distribution of RS between different time frames was compared using the Mann-Whitney *U* test, and the significance was tested at a Bonferroni-corrected *P*-value (54, 55). For each genome, a resistance pattern was calculated using the Booleanized representation of resistance in the 15 antibiotics. Thus, the pattern of 1, 1, 1, 1, 1, 1, 1, 1, 1, 1, 1, 1, 1, 1, 1 indicated resistance to all antibiotics, whereas the pattern of 0, 0, 0, 0, 0, 0, 0, 0, 0, 0, 0, 0, 0, 0, 0 indicated susceptibility to all antibiotics above. The antibiotics are ordered based on the sequence in Methods S3.

### Identification of resistant HierCC clusters

For each HierCC level, we calculated the percentage of resistant isolates in each cluster. Resistant HierCC clusters were identified when over 80% of the isolates are predicted to be resistant to a given antibiotic. From the lowest to the highest resolution HierCC level, a predicted resistant isolate is assigned to only one resistant cluster at any given HierCC level. This approach ensures that resistant clusters contain non-overlapping sets of resistant isolates.

### Hierarchical representations of resistant HierCC clusters

The hierarchical representation of resistant HierCC clusters was generated using Python (version 3.9.6) scripts from AMR analysis (53). Visualization of the hierarchical representations was performed using Graphviz’s (version 8.0.5) hierarchical layout engine (56). Human and animal host information from the NCBI database was manually simplified to represent the closest associated animal, and the top five hosts of the isolates within each cluster were displayed.

## RESULTS

### *C. jejuni* global data set

We curated a *C. jejuni* global data set of 63,102 genomes, of which 78.68% (*n* = 49,653) had country information, 67.08% (*n* = 42,347) year of collection, and 64.96% (*n* = 40,996) both year and country of collection. Genomes with both the year and country information were collected from 44 countries from 1905 to 2023, with 76.79% (*n* = 31,483) of the genomes coming from the USA and the UK. However, surveillance data from the UK ceased from 2020 onwards in the NCBI SRA database for unclear reasons; therefore, analysis of the UK data set focused on data from 2016 to 2019 (Fig. S1). The UK and the USA initiated WGS surveillance for *C. jejuni* from 2016 to 2019 and 2018 to 2022, respectively, and the USA had collected a substantial amount of *C. jejuni* WGS genomes (*n* = 6,051) from 2016 to 2017 prior to the initiation. Three datasets were constructed for downstream analysis. The UK data set included genomes from two time frames: 2016–2017 and 2018–2019. The U.S. data set consisted of genomes from three time frames: 2016–2017, 2018–2019, and 2020–2022. Lastly, the “Other” data set with genomes collected from countries outside the UK and the USA included data from three time frames: 2016–2017, 2018–2019, and 2020–2022.

### Development and characteristics of *C. jejuni* multilevel HierCC typing scheme

The UNSW *C. jejuni* cgMLST scheme core genome consisted of 1,161 core loci and a combined nucleotide length of 1.07 Mbp, which is 66.8% of the *C. jejuni* reference NCTC11168 genome length. ARI was calculated between MLST STs and clusters at each HC level to determine which HierCC level provides clustering most similar to the pubMLST MLST scheme (50) (Fig. S2). HC264 had the highest ARI with the pubMLST MLST scheme and was set as the lowest resolution level for the MHT scheme. The ARI between two nearby HierCC levels from HC0 to HC264 was calculated to identify “stable blocks” of HierCC clustering (Fig. S3). Similar to HCCeval, “stable blocks” were defined as sets of HierCC levels that define highly similar clusters (ARI ≥ 0.97) (39). Within each stable block, we identify a plateau of HierCC levels with the highest AR values. The HierCC level with the highest silhouette score with the cgMLST allelic profile distance matrix inside the plateau was selected. The final MHT scheme comprises nine levels: HC264, HC167, HC100, HC55, HC19, HC12, HC6, HC4, and HC2 (Table 1). The total number of clusters increases from HC264 to HC2. The typing resolution and discriminatory power increase as the average size of clusters becomes smaller from HC264 to HC2 (Table S8). At its highest resolution (HC2), the average size of the clusters is one, which means most of the clusters are singletons (38,152/44,148) and provides a highly discriminatory resolution for short-term epidemiology.

**TABLE 1:** Characteristics of the *C. jejuni* multilevel HierCC typing scheme

HierCC level	Number of clusters	Percentage increase of clusters from the lower resolution	Size of largest cluster (no. of genomes)	Average size of cluster	Number of singleton clusters
HC2	44,148	20%	338	1	38,152
HC4	36,785	17%	401	2	30,228
HC6	31,500	38%	521	2	24,949
HC12	22,794	29%	877	3	16,975
HC19	17,726	112%	1,142	4	12,729
HC55	8,362	66%	1,355	8	5,369
HC100	5,036	63%	2,250	13	2,955
HC167	3,099	60%	3,203	20	1,640
HC264	1,935	N/A	4,752	33	982

### Application of multilevel HierCC typing to track international movements of *C. jejuni*

**Fig 1:**
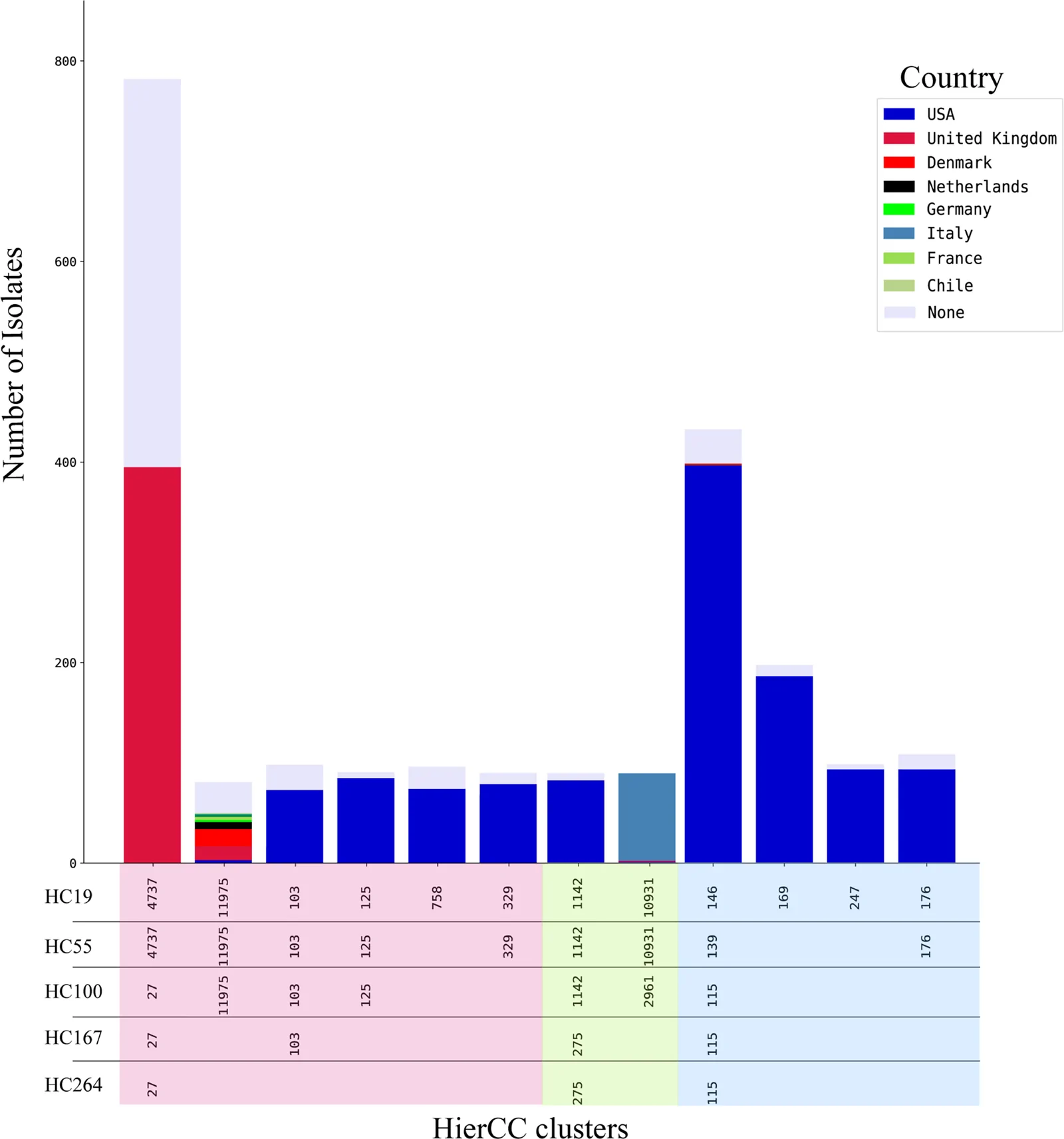
Geological diversity of *C. jejuni* in HC264 clusters 27, 275, and 115. The isolates in each column are denoted with country of origin as shown in the legend. Each column represents a unique HC19 cluster. The columns are arranged to reflect relatedness across HC264 to HC19, with columns most related to each other at the lower resolution levels appearing closer. The HierCC clusters are colored based on which HC264 cluster they belonged to in the following order: HC264 cluster 27 is colored in light red, HC264 cluster 275 is colored in light green, and HC264 115 is colored in light blue. For example, from left to right, columns 1–6 all belonged to cluster 27 at HC264. At HC167, columns 1 and 2 both belonged to HC167 cluster 27, while columns 3 to 6 belonged to HC167 cluster 103.

The MHT scheme revealed a highly diverse population structure in the *C. jejuni* global data set (Table 1). Notably, at HC264, the largest cluster (cluster 105) accounted for only 7.53% of all isolates. Among the major clusters (clusters with more than 10 genomes assigned) at different HC levels, we screened for continent-specific clusters, which were defined as a cluster with more than or equal to 85% of the genomes from one continent. HC19 was selected as the highest resolution level to examine country- and continent-specific clusters, as it is the broadest HierCC level where more than 90% of genomes were within country-specific clusters (Table S1). Different clusters exhibited distinct global distribution patterns, and more detailed country associations within the broader cluster can be revealed by further subdividing genomes at higher resolution HierCC levels. We used three clusters in HC264 (clusters 27, 275, and 115) to demonstrate the utility of the scheme to track international movements of *C. jejuni* strains (Fig. 1). For example, HC264 cluster 115 has a North America-specific distribution, consisting predominantly of isolates from the USA and only two isolates collected from Canada. The internationally distributed HC264 cluster 275 can be subdivided into cluster 1142 and cluster 2961 at HC100, which were predominantly from the USA and Italy, respectively. HC264 cluster 27 is a globally distributed cluster comprising 2,337 genomes. Within this cluster, isolates can be subdivided into two continent-specific clusters at HC167: HC167 cluster 27 for Europe and HC167 cluster 103 for North America. At HC100, the continent-specific clusters could be dissected further and identify country-specific clusters for the USA (HC100 cluster 103 and HC100 cluster 125) and the UK (HC100 cluster 27). A globally distributed HC100 cluster 11975 was also identified with 83 genomes. Most of the genomes were recovered from European countries (Germany, France, the Netherlands, Luxembourg, Denmark, and the UK), and a smaller number of genomes from the USA (*n* = 3), Egypt (*n* = 2), and Chile (*n* = 1).

### Application of multilevel HierCC typing to host attribution and transmission

Metadata analysis was performed to identify potential associations between the host types and HierCC clusters. In total, 41,076 genomes (65.1% of the total global data set) had host information and were from 49 different hosts. Among these genomes, human samples constituted 43.93% (18,047) of the genomes, whereas 53.7% (22,057 genomes) were collected from just five farm animal hosts: chicken (35%, 14,718 genomes), cattle (15.31%, 6,289 genomes), sheep (1.18%, 486 genomes), turkey (0.71%, 293 genomes), and swine (0.66%, 271 genomes). A farm animal data set was built consisting of all 22,057 genomes from chicken, cattle, sheep, swine, and turkey. In this data set, farm-animal-associated clusters are defined as clusters with more than 90% of the isolates from one type of farm animal. Using this definition, we identified HC55 as the highest resolution level to examine farm-animal-associated clusters because the majority (≥70%) of all genomes are within farm-animal-associated clusters at this level (Table S2). The farm-animal data set was combined with 18,047 human clinical genomes to identify potential host associations to human infections.

**Fig 2:**
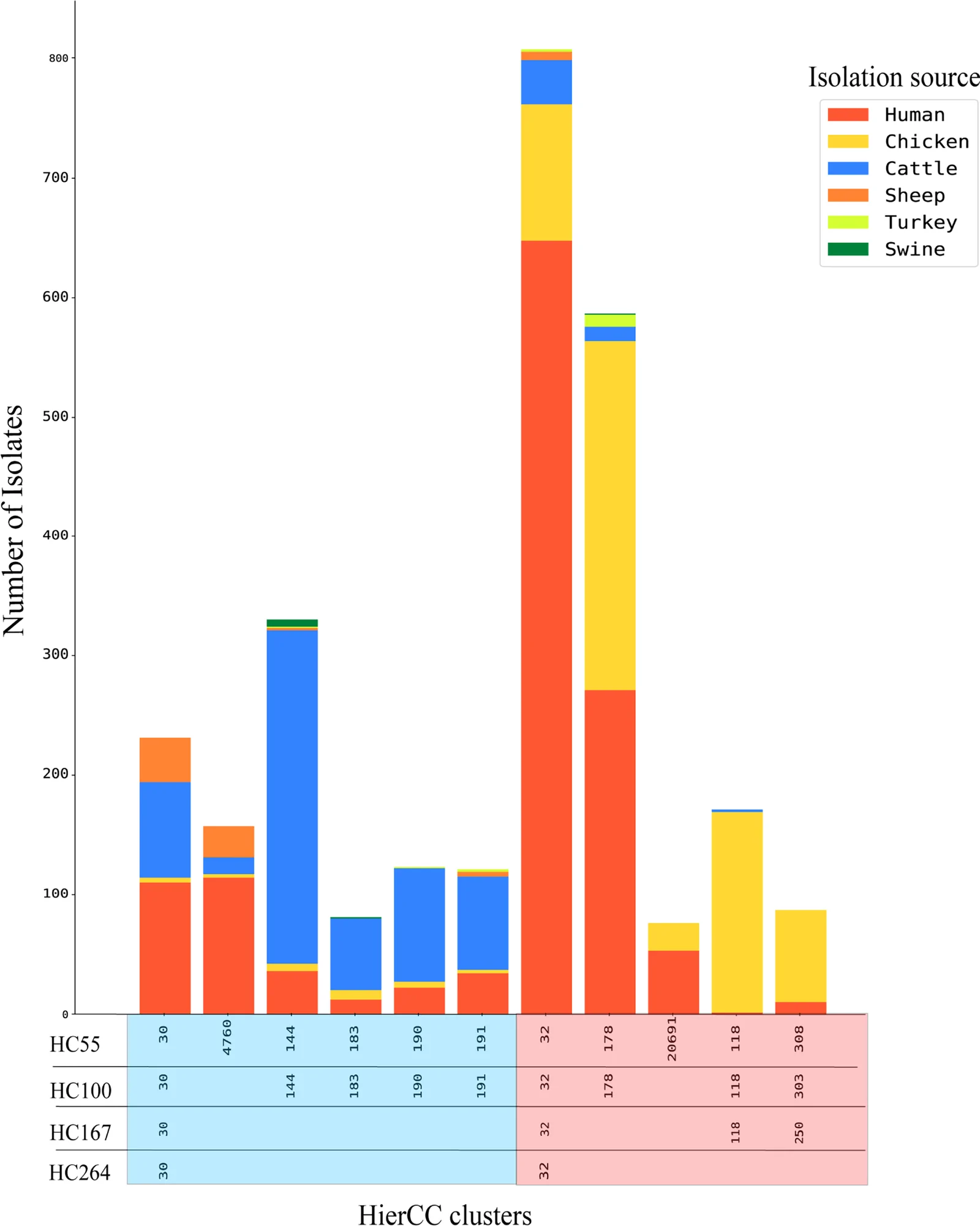
Host diversity in HC264 clusters 30 and 32. The isolates in each column are denoted with isolation source as shown in the legend. Each column represents a unique HC55 cluster. The columns are arranged to reflect relatedness across HC264 to HC55, with columns most related to each other at the lower resolution levels appearing closer. The HierCC clusters are colored based on which HC264 cluster they belonged to in the following order: HC264 cluster 30 is colored in light blue, and HC264 cluster 32 is colored in light red. For example, from left to right, columns 1–6 all belonged to cluster 30 at HC264, and at HC167, columns 1 and 2 both belonged to H100 cluster 30, while columns 3 to 6 all belonged to a unique HC100 cluster. Further examination of HC264 cluster 30 at HC100 revealed the existence of two human-cattle-sheep clusters (clusters 30 and 191) and two human-cattle clusters (clusters 183 and 190) and one human-cattle-swine cluster (cluster 144). Further partitioning of HC264 cluster 32 at HC167 identified HC167 clusters 32 and 250 with hosts of both human and chicken, and cluster 118 with only chicken as host. At HC55, HC167 cluster 32 can be further subdivided into three clusters (HC55 clusters 32, 178, and 20691). HC55 cluster 32 and 178 infect predominantly humans and chickens but also other farm animals, and HC55 cluster 20691 infects only humans and chickens. HC55 clusters 32 and 178 are generalist clusters infecting multiple animal hosts sharing the farm environment and humans. HC55 cluster 20691 is similar to HC55 cluster 308 in that they were only found in chickens and humans.

In this human and farm animal data set (*n* = 40,104), the majority of the HC264 clusters consisted of isolates from multiple host species indicating a generalist lifestyle among lineages that infect humans (57). We selected HC264 cluster 30 and 32 to demonstrate the utility of the MHT for farm animal source attribution (Fig. 2). HC264 cluster 30 showed a host preference between human and predominantly cattle. HC264 cluster 32 is a generalist cluster with 2,101 genomes, of which 55.26% and 39.08% were from humans and chickens, respectively.

### Resistance score of global *C. jejuni* isolates

Using a data set of 3,181 genomes with phenotypic antibiotic susceptibility information from the NCBI Pathogen Detection database (https://www.ncbi.nlm.nih.gov/pathogens), we assessed the accuracy of AbritAMR predicting phenotypic resistance of eight antibiotics (ampicillin, ciprofloxacin, tetracycline, azithromycin, erythromycin, gentamicin, nalidixic acid, and telithromycin) (Table S3). Overall, AbritAMR had an accuracy of 97.77% for interpreting phenotype resistance from genomic determinants in *C. jejuni*. The most common resistance determinants of each geographic location for narrow-spectrum beta-lactams, tetracyclines, quinolones, and macrolides across different countries and time frames are shown in Table S4. The complete list of resistance determinants detected by AbritAMR for the 15 antibiotic classes or combinations is shown in Table S5. A detailed overview of resistance scores in the global data set across the USA, the UK, and other countries between 2016 and 2022 is provided in Results S1.

### Resistance to medically important drug classes

**Fig 3:**
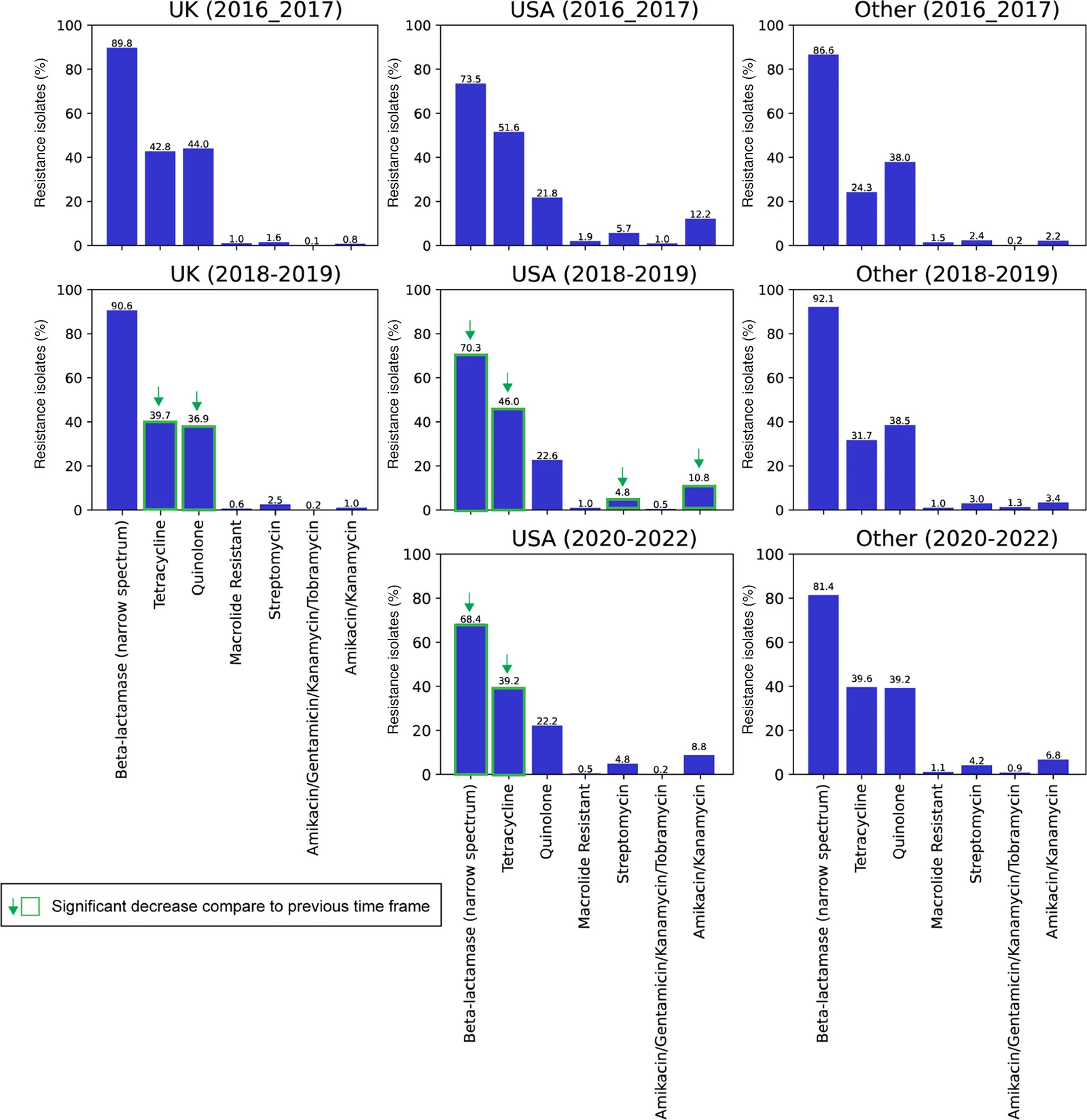
Resistant isolates to seven antibiotic classes or antibiotic combinations in the UK, the USA, and other countries. Percentage of resistant isolates to seven antibiotic classes or combinations within the local datasets of available time frames. Resistance to a given antibiotic category in different time frames was compared using the binomial test. Significance was assessed at the Bonferroni-corrected *P* < 0.0008. A significant decrease in resistance is indicated by a downward green arrow.

In the global data set, *C. jejuni* resistance to individual antibiotics varied (Fig. S4). Most notably, in descending order, 80.3% were resistant to narrow-spectrum beta-lactams, 40.4% to tetracyclines, 29.8% to quinolones, 3.4% to streptomycin, and 1.2% to macrolides. From 2016–2017 period to the 2020–2022 period, the global resistance to narrow-spectrum beta-lactams, tetracyclines, and quinolones has reduced significantly (*P* < 0.0008) (Fig. 3). Resistance to amikacin, kanamycin, and streptomycin has increased significantly from 2018 to 2019 period to the 2020–2022 period.

The resistance rate to medically important antibiotics in the UK, the USA, and other countries is shown in Fig. 3. In the UK from the 2016–2017 period to the 2018–2019 period, resistance to tetracyclines and quinolones decreased significantly. Resistance to narrow-spectrum beta-lactams remained high with no significant change during the same time frame. In the USA, resistance to narrow-spectrum beta-lactams, tetracyclines, amikacin, kanamycin, and streptomycin all significantly decreased from the 2016–2017 period to the 2018–2019 period. The decreasing trend for narrow-spectrum beta-lactams and tetracyclines continued from the 2018–2019 period to the 2020–2022 period.

### *50S_L22* mutations in the global data set

**Fig 4:**
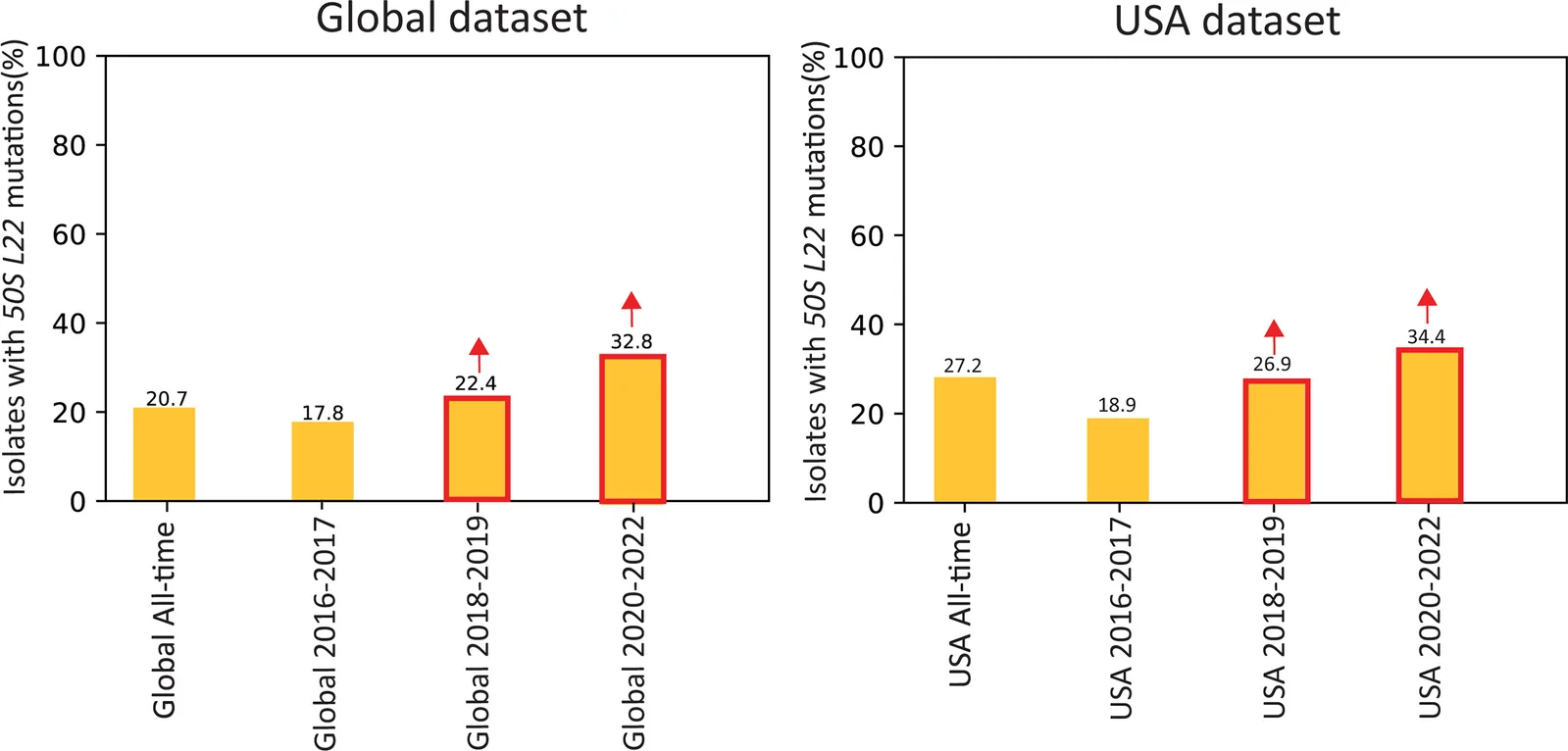
*50S_L22* mutations in the global and U.S. dataset. Percentage of isolates with *50S_L22* mutations within the datasets of available time frames. Isolates carrying the mutation in different time frames were compared using the binomial test. Significance was assessed at the Bonferroni-corrected *P* < 0.0008. Significant increases between time frames are indicated by an upward red arrow. Percentage of isolates in both the global and U.S. dataset has increased significantly from the 2016–2017 period to the 2018–2019 period, and again in the 2020–2022 period.

Although the percentage of isolates with predicted phenotypic resistance to macrolides is low in the global data set, we identified significant increases of macrolide-induced mutations in the *rplV* gene, coding for the 50S ribosomal protein L22: *50S_L22* (*A103V*) and *50S_L22* (*G86E*). In the global data set, there was a 15% increase of genomes carrying *50S_L22* gene mutations from 2016 to 2022 (Fig. 4). In the U.S. data set from 2016 to 2022, 27% (*n* = 5,866) of genomes carried *50S_L22* mutations, and there was a 15.5% increase of genomes carrying *50S_L22* mutations from the 2016–2017 period to the 2020–2022 period. We also examined the relationship between phenotypic resistance to macrolides (azithromycin and erythromycin) and the presence of both the *50S_L22 (A103V*) mutation and the *cmeABC* genes. We found 560 isolates with both *50S_L22 (A103V*) mutation and the *cmeABC* genes and phenotype resistance data in the global data set, all of which are susceptible to both azithromycin and erythromycin.

### Application of multilevel HierCC typing to identify resistant HierCC clusters and AMR surveillance

A “resistant HierCC cluster” for a given antibiotic was defined as a HierCC cluster in which at least 80% of the isolates were predicted to be resistant to that antibiotic. To ensure mutual exclusivity between resistant clusters at different HierCC levels, each resistant isolate was accounted for in only one cluster at any HierCC level. Large clusters with distinctive resistance patterns can then be further dissected at higher resolution HierCC levels to explore intricate links between AMR and HierCC clusters. The U.S. data set was chosen for the resistant HierCC cluster analysis as it is the largest data set with genomes collected from 2016 to 2022.

### Resistant HierCC clusters to medically important antibiotics in the USA from 2016 to 2022

**Fig 5:**
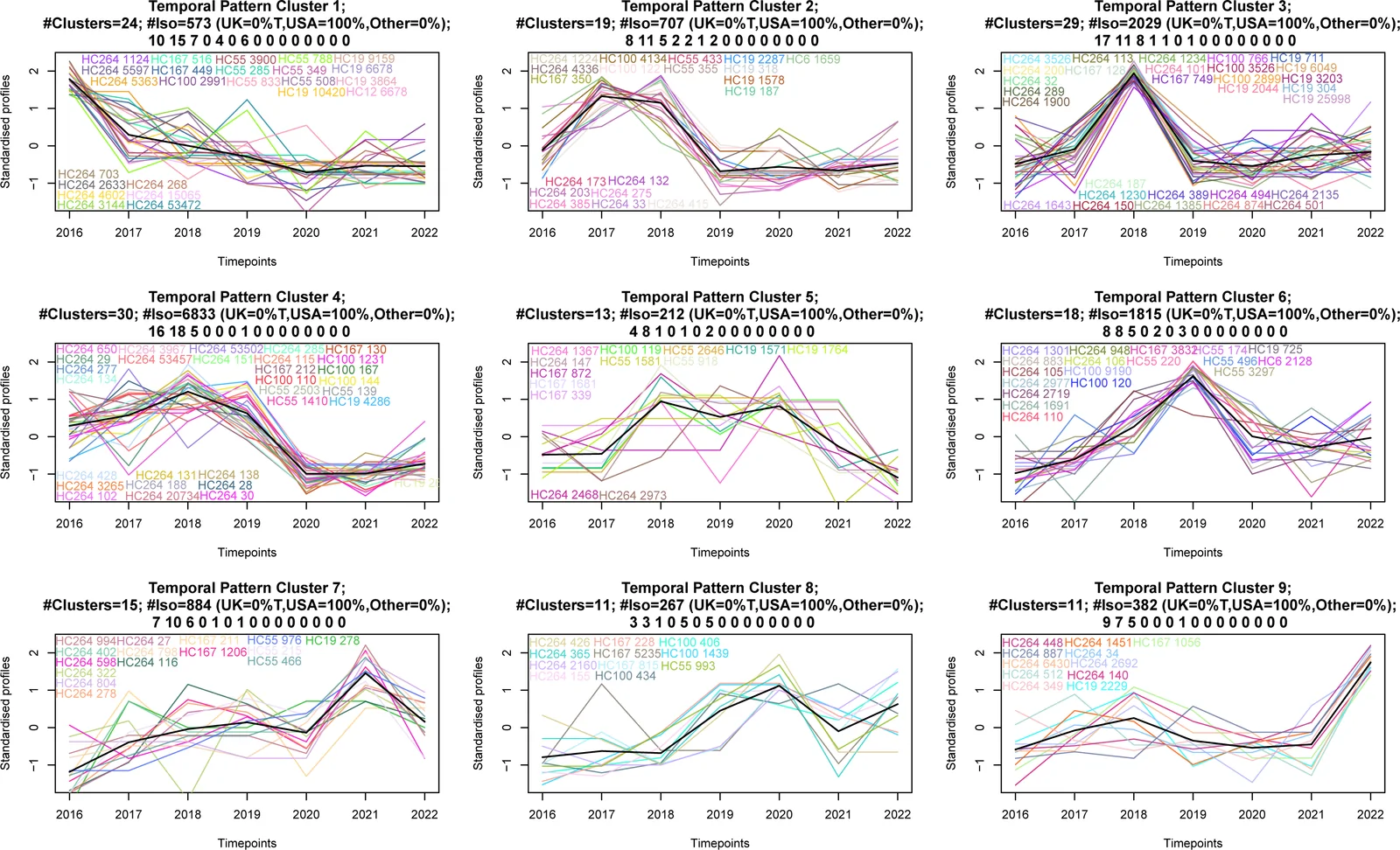
Temporal resistant pattern of 15 antibiotic classes or combinations in the USA from 2016 to 2022. Temporal trends of resistant HierCC clusters between 2016 and 2022 in the U.S. dataset. Nine TPCs contained 170 resistant clusters to 15 antibiotic classes or combinations. An individual graph with a separate header was created for each TPC. Each header indicates the number of resistant clusters (#clusters) within the TPC, total number of isolates (#Iso), and the country of origin of the isolates (UK, USA, or other). The series of numbers at the bottom of the header indicated the number of HierCC clusters resistant to a particular antibiotic class or combination (e.g., 10 15 7 0 4 0 6 0 0 0 0 0 0 0 0 in the first graph; here, 10 means 10 of the 24 clusters in this TPC are resistant to narrow-spectrum beta-lactamase). The order of the 15 antibiotic classes or combinations is as follows: (1) beta-lactamase (narrow-spectrum), (2) tetracycline, (3) quinolone, (4) macrolide, (5) streptomycin, (6) amikacin/gentamicin/kanamycin/tobramycin, (7) amikacin/kanamycin, (8) kanamycin/tobramycin, (9) amikacin/kanamycin/tobramycin, (10) lincosamides, (11) lincosamide/streptogramin, (12) chloramphenicol, (13) chloramphenicol/florfenicol, (14) sulfonamide, and (15) florfenicol/oxazolidinone.

The U.S. dataset from 2016 to 2022 was chosen to showcase the potential utility of the MHT scheme for nationwide AMR surveillance. A total of 276 resistant clusters were identified in the USA from 2016 to 2022, of which 206 resistant HierCC clusters were identified for narrow-spectrum beta-lactams, tetracyclines, quinolones, and macrolides (Table S7). We normalized the yearly abundance of resistant clusters against the average yearly count for each resistant cluster. This allowed us to group resistance clusters with similar temporal trends into temporal pattern clusters (TPCs), regardless of the size of the resistant cluster. The detailed method for identifying the TPCs is included in Methods S4. We identified historical and emerging resistant clusters from the U.S. dataset from 2016 to 2022. Nine distinctive TPCs were identified from resistant clusters of all antibiotic classes in the U.S. dataset from 2016 to 2022 (Fig. 5). The TPCs have been arranged in chronological order when the peak abundance occurred. There were 24 resistant clusters in TPC 1, all of which contained 2-fold more isolates in 2016 compared to other years, and the frequency was reduced steadily in more recent years. TPCs 2 to 7 showed groups of resistant clusters with abundance peaking at various points between 2017 and 2020, but frequency reduced significantly in more recent years. Resistant clusters in TPCs 8 and 9 showed a steep rise in abundance between 2021 and 2022, indicating emerging resistant clusters potentially associated with recent outbreaks. The temporal trends shown in Fig. 5 examine the overall resistance trends of all 15 antibiotics and classes. The complete list of resistant clusters featured in Fig. 5 is shown in Table S9. The resistance trends of narrow-spectrum beta-lactams, tetracyclines, and quinolones were examined separately and included in Results S2 and Fig. S7. We have also examined the temporal trends of *50S_L22* mutations in response to macrolides separately and included in Results S3 and Fig. S8.

### Hierarchical identification of resistant clusters in the USA from 2016 to 2022

**Fig 6:**
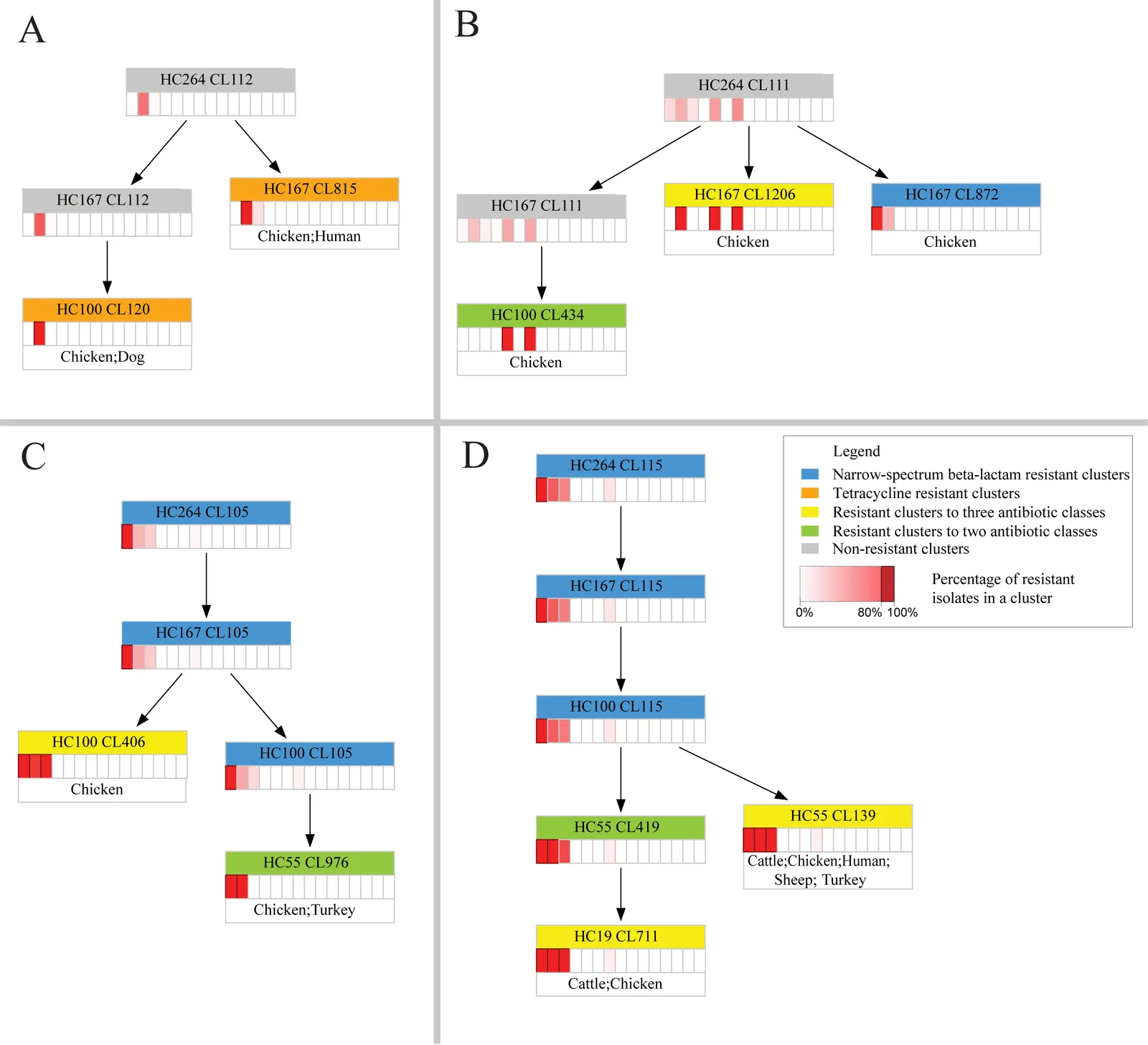
Hierarchical identification of resistant HierCC clusters in the USA from 2016 to 2022. Four scenarios of hierarchical identification of resistant HierCC clusters with at least 10 isolates in the USA from 2016 to 2022. Non-resistant clusters were colored in gray, and resistant clusters are colored based on the corresponding drug in the following order: narrow-spectrum beta-lactams (blue) and tetracyclines (orange). Clusters resistant to two antibiotics are colored in green, and clusters resistant to three antibiotics are colored in yellow. (**A**) Two tetracycline-resistant clusters. (**B**) Two streptomycin-resistant clusters and one narrow-spectrum beta-lactams resistant cluster. (**C**) Two tetracycline-resistant clusters and a series of narrow-spectrum beta-lactams-resistant clusters. (**D**) Two quinolone-resistant clusters and a series of narrow-spectrum beta-lactams-resistant clusters. The heatmap is placed at the bottom of each resistant cluster to indicate the percentage of resistant isolates to antibiotics in the following order: (1) beta-lactamase (narrow-spectrum), (2) tetracycline, (3) quinolone, (4) macrolide, (5) streptomycin, (6) amikacin/gentamicin/kanamycin/tobramycin, (7) amikacin/kanamycin, (8) kanamycin/tobramycin, (9) amikacin/kanamycin/tobramycin, (10) lincosamides, (11) lincosamide/streptogramin, (12) chloramphenicol, (13) chloramphenicol/florfenicol, (14) sulfonamide, and (15) florfenicol/oxazolidinone. A black outline indicated that at least 80% of the isolates were non-susceptible to the antibiotic at that rectangle. Beneath the heatmap, the most frequent isolation sources for the isolates in the cluster are listed.

The *C. jejuni* population in the USA can be divided into many sporadic HierCC clusters at the broadest HierCC level HC264, which was expected as the *C. jejuni* population is highly diverse (58). Hierarchical networks of resistant clusters were created to identify the acquisition of resistance in the U.S. data set, which provided novel insights into *C. jejuni* antimicrobial resistance acquisition. Four cases of hierarchical networks are presented below (Fig. 6). Scenario A depicts a nested resistant cluster to one drug at a higher resolution HierCC level inside a non-resistant cluster at a lower resolution level. HC264 cluster 112 is not a tetracycline-resistant cluster, but at higher resolution levels (HC167 and HC100), it contained two tetracycline-resistant clusters, which are HC167 cluster 815 and HC100 cluster 120 (Fig. 6A). HC167 cluster 815 was mostly isolated from chickens and humans, and HC100 cluster 120 was mostly isolated from chickens and dogs. Scenario B shows the presence of nested resistant clusters to multiple antibiotics at a higher resolution HierCC level inside a non-resistant cluster at a lower resolution level. HC264 cluster 111 is a non-resistant cluster found only in chickens but contained three resistant clusters to multiple antibiotics at HC167 and HC100 (Fig. 6B). HC167 cluster 872 was only resistant to beta-lactams. HC167 cluster 1206 was resistant to tetracyclines, streptomycin, amikacin, and kanamycin. HC100 cluster 434 was resistant to streptomycin, amikacin, and kanamycin. In scenarios C and D, nested resistant clusters to multiple drug classes or additional drugs were identified at higher resolution HC levels. HC264 cluster 105 was resistant to beta-lactams and was found in human, chicken, cattle, turkey, and wild birds (Fig. 6C). Within HC264 cluster 105, HC100 cluster 406 acquired multidrug resistance to beta-lactams, tetracyclines, and quinolones and was found only in chickens, while HC55 cluster 976 acquired additional resistance to beta-lactams and tetracyclines. Similarly, HC264 cluster 115 was resistant to beta-lactams only and found in cattle, chickens, humans, turkey, and sheep (Fig. 6D). It contained two multidrug-resistant clusters to beta-lactams, tetracyclines, and quinolones at HC55 and HC19. The two higher resolution clusters were found in different hosts, with HC19 cluster 711 found in cattle and chickens, and HC55 cluster 139 was found in cattle, cattle, chickens, human, turkey, and sheep.

## DISCUSSION

A stable and standardized genotyping scheme is essential for accurate epidemiological monitoring and AMR surveillance of *C. jejuni*. HierCC generates clusters based on the number of shared cgMLST alleles at every allelic difference level. Therefore, there is a need for guidance or standardized recommendations on the use of a subset of HierCC levels for surveillance applications, which would allow standardized communications and comparisons across time and countries. In this study, we developed a novel MHT scheme to provide standardized classification for publicly available *C. jejuni* WGS genomes worldwide (*n* = 63,102 as of June 2023). The multilevel design provided flexible typing resolutions comparable to those of traditional MLST and cgMLST and enabled the selection of appropriate typing resolutions for both short- and long-term epidemiological investigations. We used publicly available surveillance genomic data to demonstrate the utility of the *C. jejuni* MHT scheme for tracing global transmission, identifying host sources, and monitoring AMR trends.

### Comparison to the Oxford cgMLST scheme

Cody et al. designed an open-access PubMLST *C. jejuni*/*coli* cgMLST scheme consisting of 1,343 loci in v1.0 and 1,142 loci in v2.0 (50). The system was defined using both *C. coli* and *C. jejuni* strains from Oxfordshire, UK, between 2011 and 2014 (50). The UNSW *C. jejuni* cgMLST scheme was developed using *C. jejuni* isolates representing 39 countries and 2,587 MLST STs. The resulting core genome consists of 1,161 loci, which is smaller than v1.0 and larger than the v2.0 of the Oxford cgMLST scheme. The core genome of the UNSW scheme, as expected, was larger than v2.0 of the Oxford scheme, as the representative data set was comprised exclusively of *C. jejuni* genomes. Ultimately, the UNSW *C. jejuni* cgMLST scheme provides a species-specific typing scheme that captures the global diversity of *C. jejuni*.

### Epidemiologically meaningful HierCC levels selected for epidemiological applications

The European Society of Clinical Microbiology and Infectious Disease published a guideline for developing novel classification technologies for bacterial epidemiology in 2007 (59). These guidelines underline the importance of developing new typing methods that leverage readily available whole-genome data more cost-effectively. Prior to this study, hierarchical clustering of cgMLST with arbitrarily determined allelic difference levels had been applied to small *C. jejuni* data sets for AMR detection (*n* = 511) or animal source attribution (*n* = 622) (60, 61). Most notably, Hsu et al. applied the Oxford cgMLST scheme to 622 *C. jejuni* isolates and used six levels of allelic difference of 5, 10, 25, 50, 100, and 200 to demonstrate its utility to identify food animal source for human infections (61). In this study, we curated a large global data set (*n* = 63,102) of *C. jejuni* WGS genomes and identified nine epidemiologically meaningful HierCC levels (HC2, HC4, HC6, HC12, HC19, HC55, HC100, HC167, and HC264) using a statistical approach similar to HCCeval (39). The epidemiologically meaningful levels provided a standardized recommendation of core genome allelic distances for surveillance applications, which would allow standardized communications and comparisons across time and regions. By integrating the nine levels into one comprehensive multilevel scheme, the MHT scheme provides a standardized hierarchy for related strains with flexible typing resolution. Strains are grouped into larger clusters at broader HierCC levels and smaller clusters as the resolution increases (Table 1). Because clusters at different HierCC levels contained a hierarchical set of genomes, the higher resolution clusters could be examined in the context of their lower resolution clusters to provide epidemiological and evolutionary inferences. Broader HierCC levels from HC264 to HC19 are useful for investigating longer-term and global epidemiology, while higher resolution levels from HC12 to HC2 can provide similar discriminatory power to cgMLST schemes for outbreak detection and other short-term applications. We demonstrated the potential utility of the scheme for epidemiological surveillance at local and global levels. We identified farm-animal-associated clusters up to HC55, country- and continent-specific clusters up to HC19, and utilized the hierarchical network between the HierCC levels to track emergence and persistence of AMR genotypes.

### Multilevel HierCC typing scheme allows tracking of *C. jejuni* global distribution

Large numbers of livestock and animal products are traded each year across the globe in the age of globalization, facilitating the spread of foodborne pathogens (62). By identifying country- and continent-specific clusters at each HierCC level, the multilevel HierCC scheme allows genomes with sporadic occurrence to be grouped into larger, more informative clusters. This utilization can potentially provide useful information for tracking the movement of pathogenic *C. jejuni* strains beyond borders and identifying international outbreak clusters. HC264 cluster 275 contained genomes from routine surveillance between 2013 and 2022 from the USA and genomes from a single campylobacteriosis outbreak (63, 63). At HC100, the Italian outbreak cluster (HC100 cluster 2961) could be differentiated from the U.S.-specific cluster (HC100 cluster 1142) (Fig. 1). The connection between the U.S.-specific cluster and the Italian outbreak would have been overlooked by traditional single-level typing scheme with fixed resolution.

### Multilevel HierCC typing scheme provides flexible typing resolution for *C. jejuni* farm animal host attribution

Based on preference to a single host or multiple hosts, *C. jejuni* lineages can be categorized as specialist or generalist, respectively (64). However, methods used to identify host-associated *C. jejuni* groups are inconsistent between studies using conventional MLST or its clonal complex-based typing system (64, 65). Compared with these conventional methods, the flexibility of the MHT scheme provided the appropriate typing resolution to identify the most likely farm animal source of human infections with much higher precision (Fig. 2). The cluster nomenclature remained consistent at each HierCC level, while the flexible typing resolution allows for differentiation between specialist and generalist genome types. This could potentially provide more accurate source attribution and aid a more targeted approach for prevention and control.

### Antibiotic resistance in the global data set

In the complete global data set, over 90% of the isolates were predicted to be resistant to at least one antibiotic. The high overall resistance rate in the global *C. jejuni* population can potentially be linked to the heavy reliance on antimicrobials to maintain health and productivity during intensive farm animal production (18, 66–68). Variations in regulations and legislation across countries can result in differing antibiotic selection pressures. Therefore, it is essential to analyze the global data set by country of origin. The UK and the USA introduced WGS surveillance programs for *C. jejuni* in 2016 and 2018, respectively (25, 26). These programs generated large volumes of high-quality WGS data in the public domain, making them particularly valuable for AMR surveillance investigations.

In the UK, an AMR national action plan reduced the usage of antibiotics in humans and animals in the UK by 28% from 2014 to 2020 (69–71). Correspondingly, our analysis of the genome data found that while resistance to narrow-spectrum beta-lactams remained high, resistance to tetracyclines and quinolones significantly decreased from 2016–2017 to 2018–2019.

In the USA, proportions of isolates resistant to beta-lactams and tetracycline have decreased significantly, while resistance to quinolones remained stable in the USA from 2016 to 2022. Although resistance to macrolide remained low (~2%) in both the UK and the USA, proportions of genomes with *50S_L22* mutations have increased significantly in the USA from 2016 to 2022. According to the FDA, the sale of medically important antibiotics for veterinary uses in the USA had increased by more than 8% from 2017 to 2020 (19). The widespread use of tylosin, a macrolide antibiotic, in cattle and chicken feed in the U.S. agricultural industry could be a factor contributing to the increase of *50S_L22* mutations (19, 66–68, 72, 73).

For countries outside of the UK and the USA without a WGS surveillance program, the genomes were combined to show the prevalence of resistance in each time frame. Overall, predicted resistance in the other countries fluctuated from 2016 to 2022, which could be due to the sampling bias toward outbreak strains in these countries, as no countries other than the USA and the UK were actively monitoring *C. jejuni*. Although it is important to present the AMR prediction results for the other countries, we refrain from discussing the significance of these results due to the heterogeneous nature of the data set. This highlights the importance of consistent and continuous WGS surveillance programs for *C. jejuni* to provide AMR prediction and tracking at local, continental, and global scales.

### Temporal trends and hierarchical network of resistant HierCC clusters to medically important antibiotics in the USA from 2016 to 2022

Using the USA 2016–2022 surveillance data set, we demonstrated the utility of the MHT scheme for identifying and monitoring *C. jejuni* resistance to medically important antibiotic classes. *C. jejuni* can rapidly transfer resistance genes between strains via natural transformation or conjugation (74, 75). The MHT scheme can provide the ideal typing resolution to identify clusters associated with antibiotic resistance. Each cluster can be consistently identified across time, allowing continuous monitoring of strains with specific resistance patterns. Strains with similar resistance patterns at broader levels often group together at higher resolution levels. The hierarchical structure enabled rapid identification of resistant clusters by subsetting closely related strains with similar resistance patterns at higher resolution HierCC levels through network analysis.

The application of clustering resistant clusters based on their temporal trend patterns is particularly useful for pathogens with sporadic outbreak patterns such as *C. jejuni*, as not all resistant clusters are associated with recent outbreaks. In the U.S. data set, although decreased resistance rates for beta-lactams and tetracyclines were observed from 2016 to 2022, several resistant HierCC clusters containing hundreds of resistant isolates have shown 1.5- to 2-fold increases in frequency from 2020 to 2022 (Fig. S5 and S6). The majority of the recent emerging resistant clusters carried *bla*_OXA_ genes coding for beta-lactam resistance and the *tetO* gene for tetracycline resistance. These clusters have become dominant in more recent years, which would have been ignored by traditional tracking methods. The MHT scheme provides a means to differentiate and track these clusters, pinpointing emerging resistance and the genetic background of the strains from which the resistance arose. It enables precise surveillance of resistant clusters and better design of targeted prevention and control strategies.

We further identified sequential acquisition of resistance using network analysis. A subset of beta-lactam-resistant clusters at HC264 acquired tetracycline resistance genes and evolved into resistance to multiple antibiotics as resistant clusters at higher HierCC levels (Fig. 6C). Similarly, the prevalence of quinolone resistance in *C. jejuni* was around ~21% in the U.S. data set with the most prevalent resistant mechanism detected being mutations in *gyrA*. Our hierarchical network analysis found that clusters resistant to other antibiotics can acquire resistance to quinolones rapidly. As shown in Fig. 6D, two subsets of narrow-spectrum beta-lactam-resistant HC264 cluster 1115 (HC55 cluster 139 and HC19 cluster 711) acquired additional resistance to quinolones. The MHT scheme had provided a more targeted platform for analyzing emergence and changing trends of resistance.

### Monitoring *50S_L22* mutations in response to selection pressure of macrolides

In *C. jejuni*, phenotypic macrolide resistance is mostly associated with *erm*(B) and mutations in the 23S rRNA (*75–77). 50S_L22* mutations alone do not confer phenotypic macrolide resistance in *C. jejuni* (78). Previous studies have reported that the synergy between the efflux pump CmeABC and the *50S_L22(A103V*) mutation may lead to resistance to macrolides in *C. jejuni* (79). However, by utilizing phenotypic data from the NCBI Pathogen Detection database, we have found that all the isolates carrying both the *cmeABC* genes and the *50S_L22(A103V*) mutation are susceptible to both azithromycin and erythromycin. Nonetheless, *50S_L22* mutations serve as a reliable marker of macrolide selection pressure (80). However, mutation of the *50S_L22* gene downregulates motility and metabolism genes, which is a significant fitness cost under no selection pressure for mutated strains to compete with strains without the mutation (81).

By utilizing the temporal trend analysis, we identified *50S_L22* mutation-carrying clusters correlated with the increase of macrolide usage at specific time frames (Fig. S8). The increased percentage of genomes carrying *50S_L22* mutations from 2016 to 2022 indicated elevated selection pressure from macrolide usage in the USA. This can be correlated with the widespread usage of macrolides in the U.S. chicken and cattle farming industry (19, 67, 73). In the USA, *50S_L22* mutations increased significantly from the 2016–2017 period to the 2018–2019 period, and again from the 2018–2019 period to the 2020–2022 period (Fig. 4). The MHT scheme enables continuous tracking and monitoring of AMR or AMR-associated mutations for better-informed agricultural legislation and public health safety.

### Conclusions

In this study, we built a multilevel HierCC typing scheme to provide flexible, stable, and standardized genomic typing for *C. jejuni*. We formulated a statistical process to identify epidemiologically meaningful HierCC levels for both long- and short-term epidemiological analyses. The multilevel design provided flexible typing resolution while maintaining the stability of the overall nomenclature. The nine epidemiologically meaningful levels also provided a reference for comparison between single-level scheme-based local studies.

The scheme has shown promising utility in identifying continent- or country-specific clusters and farm-animal-specific clusters in *C. jejuni* populations. Importantly, the scheme enabled tracking and monitoring of AMR trends to medically important antibiotic classes. Using surveillance genome data from the USA and the UK, we showcased the robustness of the scheme in AMR surveillance. We believe that the MHT scheme has the potential to be integrated into a continuous and comprehensive *C. jejuni* surveillance program, supporting data-driven decision-making for the prevention and control of *C. jejuni* globally.

## Data Availability

A guide to download and implement the UNSW *C. jejuni* cgMLST scheme and the *C. jejuni* multilevel HierCC typing scheme has been created and is freely available at GitHub (https://github.com/LanLab/C.jejuni-multilevel-HierCC-typing-scheme).

## SUPPLEMENTAL MATERIAL

Supplemental material 1 aac.00310-26-s0001.docx Supplemental methods.

Supplemental material 2 aac.00310-26-s0002.docx Supplemental results.

Supplemental figures aac.00310-26-s0003.docx Fig. S1 to S8.

Supplemental tables aac.00310-26-s0004.docx Tables S1 to S9.

ASM does not own the copyrights to Supplemental Material that may be linked to, or accessed through, an article. The authors have granted ASM a non-exclusive, world-wide license to publish the Supplemental Material files. Please contact the corresponding author directly for reuse.
