# Supplementary material for: Multilevel HierCC typing scheme for global genomic epidemiology and antimicrobial resistance surveillance of Campylobacter jejuni
Source: Antimicrob Agents Chemother. 2026 Jul 28;70(9):e00310-26. doi: 10.1128/aac.00310-26 (PMC13713005; doi:10.1128/aac.00310-26)
Supplement: Supplementary file 1 [file aac.00310-26-s0001.docx]

**Supplementary Methods**

**S1 Core loci validation for the global *C. jejuni* cgMLST**

The assembled genomes were filtered based on the following quality thresholds: genomes size between 1509232 bp and 1858564 bp, a maximum of 119 contigs, a maximum of 292243 bp for N50, and a maximum L50 of 16.5 contigs.

**S2 Core loci validation for the global *C. jejuni* cgMLST**

We downloaded 346 publicly available *C. jejuni* complete whole genome sequences from NCBI (<https://www.ncbi.nlm.nih.gov/>) and a training file generated by Prodigal v2.6.3 from the *C. jejuni* reference genome (RefSeq Accession GCF_000304375.1) to establish the initial schema seeds for the global *C. jejuni* global cgMLST scheme. The Comprehensive and Highly Efficient Workflow BSR-Based Allele Calling Algorithm (chewBBACA v3.3.2) predicted 3920 coding sequences (CDS) from the 346 complete genomes. Allele calling was performed with a Blast Score Ratio of 0.6 to redetermine the core loci from the schema seeds. For a CDS to be considered a core locus, it must be present in >=95% of the diverse representative dataset (n=2587).

**S3 Order of the antibiotics and antibiotic classes in resistance score**

1. beta-lactamase (narrow-spectrum), 2. tetracycline, 3. quinolone, 4. macrolide, 5. streptomycin, 6. amikacin/gentamicin/kanamycin/tobramycin, 7. amikacin/kanamycin, 8. kanamycin/tobramycin, 9. amikacin/kanamycin/tobramycin, 10. lincosamides, 11. lincosamide/streptogramin, 12. chloramphenicol, 13. chloramphenicol/florfenicol, 14. sulfonamide, 15. florfenicol/oxazolidinone.

**S4 Identification of TPC of resistant HierCC clusters**

The temporal patterns of resistant HierCC clusters were determined using similar methods as described by Kaur *et al*. [1]. Briefly, the sampling frequency of each resistant cluster from a given antibiotic were normalised based on the average yearly count for that cluster. The normalization was performed in python (version 3.9.6). The resistant clusters to 15 antibiotics or antibiotic combinations were then clustered in TPCs based on similarities in temporal abundance patterns to determine the timing of their emergence and changes in sampling frequency over time. The optimal number of TPCs for each dataset was determined through a combination of the silhouette index and manual inspection of the temporal delineation of the clusters. The silhouette index was calculated using the R package Bios2mdS (version 1.2.3) [2]. TPCs were then generated using the unsupervised c-means clustering algorithm in the Mfuzz (version 2.60.0) package of R (version 4.3.1) [3].

**References**

**1. Kaur S, Payne M, Partridge SR, Sintchenko V, Lan R. A dissection of the genomic antimicrobial resistance epidemiology of *Salmonella* Typhimurium. 2024; https://doi.org/10.1101/2024.05.12.593721**

**2. Pelé J, Bécu J-M, Abdi H, Chabbert M. Bios2mds: an R package for comparing orthologous protein families by metric multidimensional scaling. BMC Bioinformatics. 2012;13:133. https://doi.org/10.1186/1471-2105-13-133**

**3. Kumar L, E Futschik M. Mfuzz: a software package for soft clustering of microarray data. Bioinformation. 2007;2:5–7. https://doi.org/10.6026/97320630002005**
