# Supplementary material for: Multilevel HierCC typing scheme for global genomic epidemiology and antimicrobial resistance surveillance of Campylobacter jejuni
Source: Antimicrob Agents Chemother. 2026 Jul 28;70(9):e00310-26. doi: 10.1128/aac.00310-26 (PMC13713005; doi:10.1128/aac.00310-26)
Supplement: Supplementary file 2 [file aac.00310-26-s0002.docx]

**Supplementary Results**

**S1. Detailed overview of resistance scores in the global dataset.**

The resistance scores in the global dataset across the USA, the UK, and other countries between 2016 and 2022 is presented in **Supplementary Tables: Table S6**. In total, only 8.27% of the isolates were predicted to be susceptible to all the antibiotics and majority (46.58%) of the isolates were predicted to be resistant to only one drug class. In the USA, the overall resistance remained stable and decreased 1.91% from 2016-2017 to 2018-2019 and decreased further by 1.81% from 2018-2019 to 2020-2022. Resistance to one antibiotic class has increased significantly by 4.7% in the USA from 2016 to 2022. In the UK, the overall resistance percentage in the UK remained stable from 2016-2017 to 2018-2019, but the average proportion of susceptible isolates was lower (~3.5%) compared to the USA (~12%) in the same time period. The resistance score also allowed identification of isolates resistant to multiple antibiotics and antibiotic classes in the global dataset (**Supplementary Tables: Table S6**). In total, 24.4% of the isolates were resistant to two drug classes, 17.98% of the isolates were resistant to three drug classes and 1.83 % of the isolates resistant to four drug classes.

**S2. Temporal trends of resistance to medically important antibiotics in the USA from 2016-2022**

**S2.1 Narrow-spectrum beta-lactams**

Temporal trends of beta-lactam resistant clusters in the USA from 2016-2022 are shown in **Supplementary Figures: Figure S5**. Overall resistance to beta-lactams in the USA decreased by 5.1% from the 2016-17 period to the 2020-2022 period (**Figure 3**). The decrease in overall resistant correlated to the temporal trends in TPC 1-7, where sampling frequency of 67 resistant clusters in these TPCs fell below average in the 2020-2022 period. Despite overall decrease in the percentage of beta-lactam resistant strains in the USA dataset from the 2016-17 period to the 2020-2022 period, 15 resistant clusters in TPC 8-9 showed on average 1.5 to 2-fold increase in frequency in the 2020-2022 period.

**S2.2Tetracyclines**

Temporal trends of tetracycline resistant clusters in the USA from 2016-2022 are shown in **Supplementary Figures: Figure S6**. TPC 1-7 representing 69 tetracycline resistant clusters had reduced frequency in the 2020-2022 period, falling below average. Resistant clusters in TPC 8 showed small increase in frequency since 2020 and peaked at 1-fold increase in 2021. TPC 9 had 7 resistant clusters with 2-fold increase in frequency in 2022.

**S2.3Quinolones**

Temporal trends of quinolone resistant clusters in the USA from 2016-2022 are shown in **Supplementary Figures: Figure S7**. Percentage of isolates resistant to quinolones remained stable in the USA from 2016-2022 (**Figure 3**). Sampling frequency of TPC 3, 4 and 5 peaked at year 2018, 2019 and 2021 respectively, and TPC 6 with 5 resistant clusters showed 2-fold increase in sampling frequency in 2022.

**S3. Temporal trends of *50s_L22* mutation in response to macrolides**

Considering that macrolides (azithromycin and erythromycin) are medically important antibiotics, tracking of temporal trends in *50s_L22* mutations is crucial for identifying and monitoring underlying selection pressure. To achieve this, “*50S_L22* mutation HierCC cluster” was defined as a HierCC cluster in which at least 80% of the genomes were carrying *50S_L22* mutations. Temporal patterns of *50S_L22* mutation clusters in the USA from 2016-2022 are shown in **Supplementary Figures: Figure S8**. Frequency of *50S_L22* mutation clusters in TPC 1-3 peaked prior to 2020 and gradually decreased afterwards. Frequency of *50S_L22* mutation clusters in TPC 4-5 has peaked prior to the 2020-2022 period and remain stable in more recent years. TPC 6 contained three *50S_L22* mutation clusters with 1.5-fold increase in frequency between 2020-2022.
