# Supplementary material for: Multilevel HierCC typing scheme for global genomic epidemiology and antimicrobial resistance surveillance of Campylobacter jejuni
Source: Antimicrob Agents Chemother. 2026 Jul 28;70(9):e00310-26. doi: 10.1128/aac.00310-26 (PMC13713005; doi:10.1128/aac.00310-26)
Supplement: Supplementary file 4 [file aac.00310-26-s0004.docx]

**Supplementary Tables**

**Table S1 Metrics of continent-specific clusters at different HierCC levels**

| HierCC level | *Percentage of major cluster isolates in continent-specific clusters | *Number of major cluster isolates in continent specific clusters | Number of isolates in major clusters (>=10 isolates) | Number of continent-specific clusters |
| --- | --- | --- | --- | --- |
| HC2 | 93.39% | 8501 | 9103 | 308 |
| HC4 | 95.22% | 14371 | 15093 | 466 |
| HC6 | 94.71% | 19392 | 20476 | 592 |
| HC12 | 95.85% | 29135 | 30395 | 731 |
| HC19 | 93.38% | 34549 | 36998 | 749 |
| HC55 | 88.02% | 44012 | 50002 | 593 |
| HC100 | 83.95% | 46022 | 54823 | 425 |
| HC167 | 63.17% | 36523 | 57817 | 302 |
| HC264 | 45.46% | 27173 | 59779 | 182 |

***Accounting for only isolates from major clusters (>=10 isolates)**

**Table S2 Metrics of Host-specific clusters at different HierCC levels**

| HierCC level | *Percentage of major cluster isolates in farm-animal-associated clusters | Number of farm-animal-associated clusters | *Number of major cluster isolates in farm-animal-associated clusters | Number of isolates in major cluster (>=10) isolates clusters |
| --- | --- | --- | --- | --- |
| HC2 | 0.8914 | 62 | 1247 | 1399 |
| HC4 | 0.8356 | 103 | 2449 | 2931 |
| HC6 | 0.7644 | 137 | 3351 | 4384 |
| HC12 | 0.7192 | 197 | 5270 | 7328 |
| HC19 | 0.7062 | 239 | 6952 | 9844 |
| HC55 | 0.7052 | 259 | 11307 | 16034 |
| HC100 | 0.6975 | 208 | 12761 | 18296 |
| HC167 | 0.6207 | 150 | 12299 | 19814 |
| HC264 | 0.5405 | 94 | 11252 | 20817 |

***Accounting for only isolates from major clusters (>=10 isolates)**

**Table S3 Accuracy of AbritAMR phenotype interpretation against *C. jejuni***

| Antibiotic | Phenotype Resistance | AbritAMR interpretation of resistance | Accuracy |
| --- | --- | --- | --- |
| ampicillin | 21 | 21 | 100.00% |
| ciprofloxacin | 658 | 653 | 99.24% |
| tetracyclines | 1528 | 1509 | 98.76% |
| azithromycin | 75 | 74 | 98.67% |
| nalidixic acid | 634 | 624 | 98.42% |
| erythromycin | 78 | 76 | 97.44% |
| gentamicin | 29 | 28 | 96.55% |
| telithromycin | 58 | 54 | 93.10% |
| **Average** |  |  | **97.77%** |

| Drug class | UK 2016-2019 | USA 2016-2022 | Others 2016-2022 |
| --- | --- | --- | --- |
| Narrow-spectrum beta-lactams | *bla*_OXA-193_ (60.12%), *bla*_OXA-461_ (7.66%), *bla*_OXA-184_ (7.62%), bla_OXA-61_ (6.80%) | *bla*_OXA-193_ (58.54%), *bla*_OXA-184_ (8.37%), *bla*_OXA-61_ (6.91%) | *bla*_OXA-193_ (59.52%), *bla*_OXA-461_ (5.94%), *bla*_OXA-61_ (5.03%) |
| Tetracyclines | *tetO* (99.37%) | *tetO* (99.67%) | *tetO* (97.86%) |
| Quinolones | *gyrA*_T86I (97.49%) | *gyrA*_T86I (98.58%) | *gyrA*_T86I (94.29%) |
| Macrolides | *23S*_A2075G (88.89%), *23S*_A2074T (3.70%) | *23S*_A2075G 90.99%), *23S*_A2074T (6.01%) | *23S*_A2074T (42.86%), *23S*_A2075G (35.71%) |

**Table S4 Most common resistance determinants for UK, USA and other countries**

**Table S5 Resistance genes and mutations detected by AbritAMR in the global dataset**

*R_score: resistance score indicating the number of antibiotics to which an isolate was predicted to be resistant.

**Table S6 Resistance score of the global dataset, the UK, the USA, and other countries from 2016-2022**

**Table S7 Resistant clusters to medically important antibiotics in the USA from 2016-2022.**

| Drug | HC264 | HC167 | HC100 | HC55 | HC19 | HC12 | HC6 | HC4 | HC2 | 2016-2022 resistant clusters |
| --- | --- | --- | --- | --- | --- | --- | --- | --- | --- | --- |
| Beta-lactams (narrow-spectrum) | 78 | 2 | 0 | 1 | 0 | 0 | 0 | 0 | 0 | 81 |
| Tetracyclines | 27 | 11 | 12 | 15 | 19 | 0 | 2 | 0 | 0 | 86 |
| Quinolones | 23 | 1 | 2 | 4 | 5 | 1 | 0 | 0 | 0 | 36 |
| Macrolide | 1 | 0 | 1 | 0 | 1 | 0 | 0 | 0 | 0 | 3 |
| Total |  |  |  |  |  |  |  |  |  | **206** |

**Table S8*.*** **Characteristics of nine *C. jejuni* HierCC levels**

| HierCC level | Number of clusters | Percentage increase of clusters from the lower resolution HierCC level | Size of largest cluster (no. of genomes) | Average size of cluster (rounded down to whole numbers) | Number of singleton clusters |
| --- | --- | --- | --- | --- | --- |
| HC2 | 44148 | +20% | 338 | 1 | 38152 |
| HC4 | 36785 | +17% | 401 | 2 | 30228 |
| HC6 | 31500 | +38% | 521 | 2 | 24949 |
| HC12 | 22794 | +29% | 877 | 3 | 16975 |
| HC19 | 17726 | +112% | 1142 | 4 | 12729 |
| HC55 | 8362 | +66% | 1355 | 8 | 5369 |
| HC100 | 5036 | +63% | 2250 | 13 | 2955 |
| HC167 | 3099 | +60% | 3203 | 20 | 1640 |
| HC264 | 1935 | The lowest level of resolution | 4752 | 33 | 982 |

**Table S9*.*** **List of resistant clusters in the temporal pattern clusters in Figure 5**

| HierCC clusters | Resistance | Temporal Pattern Cluster (TPC) |
| --- | --- | --- |
| HC100 2991 | Tetracycline | 1 |
| HC12 6678 | Quinolone | 1 |
| HC167 449 | Tetracycline | 1 |
| HC167 516 | Tetracycline | 1 |
| HC19 10420 | Tetracycline | 1 |
| HC19 3864 | Tetracycline | 1 |
| HC19 6678 | Tetracycline | 1 |
| HC19 9159 | Tetracycline | 1 |
| HC264 1124 | Beta-lactamase (narrow spectrum) | 1 |
| HC264 15065 | Beta-lactamase (narrow spectrum) | 1 |
| HC264 2633 | Beta-lactamase (narrow spectrum) | 1 |
| HC264 268 | Beta-lactamase (narrow spectrum) | 1 |
| HC264 3144 | Beta-lactamase (narrow spectrum) | 1 |
| HC264 4602 | Beta-lactamase (narrow spectrum) | 1 |
| HC264 53472 | Beta-lactamase (narrow spectrum) | 1 |
| HC264 5363 | Beta-lactamase (narrow spectrum) | 1 |
| HC264 5597 | Beta-lactamase (narrow spectrum) | 1 |
| HC264 703 | Beta-lactamase (narrow spectrum) | 1 |
| HC55 285 | Tetracycline | 1 |
| HC55 349 | Quinolone | 1 |
| HC55 3900 | Tetracycline | 1 |
| HC55 508 | Tetracycline | 1 |
| HC55 788 | Tetracycline | 1 |
| HC55 833 | Streptomycin | 1 |
| HC100 122 | Tetracycline | 2 |
| HC100 4134 | Macrolide Resistant | 2 |
| HC167 350 | Streptomycin | 2 |
| HC19 1578 | Tetracycline | 2 |
| HC19 187 | Tetracycline | 2 |
| HC19 2287 | Tetracycline | 2 |
| HC19 318 | Macrolide Resistant | 2 |
| HC264 1224 | Beta-lactamase (narrow spectrum) | 2 |
| HC264 132 | Tetracycline | 2 |
| HC264 173 | Beta-lactamase (narrow spectrum) | 2 |
| HC264 203 | Beta-lactamase (narrow spectrum) | 2 |
| HC264 275 | Beta-lactamase (narrow spectrum) | 2 |
| HC264 33 | Beta-lactamase (narrow spectrum) | 2 |
| HC264 385 | Beta-lactamase (narrow spectrum) | 2 |
| HC264 415 | Beta-lactamase (narrow spectrum) | 2 |
| HC264 4336 | Beta-lactamase (narrow spectrum) | 2 |
| HC55 355 | Tetracycline | 2 |
| HC55 433 | Tetracycline | 2 |
| HC6 1659 | Tetracycline | 2 |
| HC100 2899 | Streptomycin | 3 |
| HC100 3526 | Tetracycline | 3 |
| HC100 766 | Tetracycline | 3 |
| HC167 128 | Tetracycline | 3 |
| HC167 749 | Beta-lactamase (narrow spectrum) | 3 |
| HC19 2044 | Tetracycline | 3 |
| HC19 25998 | Tetracycline | 3 |
| HC19 304 | Tetracycline | 3 |
| HC19 3203 | Tetracycline | 3 |
| HC19 6049 | Quinolone | 3 |
| HC19 711 | Quinolone | 3 |
| HC264 101 | Beta-lactamase (narrow spectrum) | 3 |
| HC264 113 | Beta-lactamase (narrow spectrum) | 3 |
| HC264 1230 | Beta-lactamase (narrow spectrum) | 3 |
| HC264 1234 | Beta-lactamase (narrow spectrum) | 3 |
| HC264 1385 | Beta-lactamase (narrow spectrum) | 3 |
| HC264 150 | Beta-lactamase (narrow spectrum) | 3 |
| HC264 1643 | Macrolide Resistant | 3 |
| HC264 187 | Beta-lactamase (narrow spectrum) | 3 |
| HC264 1900 | Beta-lactamase (narrow spectrum) | 3 |
| HC264 200 | Beta-lactamase (narrow spectrum) | 3 |
| HC264 2135 | Beta-lactamase (narrow spectrum) | 3 |
| HC264 289 | Beta-lactamase (narrow spectrum) | 3 |
| HC264 32 | Beta-lactamase (narrow spectrum) | 3 |
| HC264 3526 | Beta-lactamase (narrow spectrum) | 3 |
| HC264 389 | Beta-lactamase (narrow spectrum) | 3 |
| HC264 494 | Tetracycline | 3 |
| HC264 501 | Beta-lactamase (narrow spectrum) | 3 |
| HC264 874 | Beta-lactamase (narrow spectrum) | 3 |
| HC100 110 | Tetracycline | 4 |
| HC100 1231 | Tetracycline | 4 |
| HC100 144 | Tetracycline | 4 |
| HC100 167 | Quinolone | 4 |
| HC167 130 | Tetracycline | 4 |
| HC167 212 | Tetracycline | 4 |
| HC19 2609 | Tetracycline | 4 |
| HC19 4286 | Tetracycline | 4 |
| HC264 102 | Beta-lactamase (narrow spectrum) | 4 |
| HC264 115 | Beta-lactamase (narrow spectrum) | 4 |
| HC264 131 | Beta-lactamase (narrow spectrum) | 4 |
| HC264 134 | Beta-lactamase (narrow spectrum) | 4 |
| HC264 138 | Beta-lactamase (narrow spectrum) | 4 |
| HC264 151 | Beta-lactamase (narrow spectrum) | 4 |
| HC264 188 | Tetracycline | 4 |
| HC264 20734 | Beta-lactamase (narrow spectrum) | 4 |
| HC264 277 | Tetracycline | 4 |
| HC264 28 | Beta-lactamase (narrow spectrum) | 4 |
| HC264 285 | Beta-lactamase (narrow spectrum) | 4 |
| HC264 29 | Tetracycline | 4 |
| HC264 30 | Beta-lactamase (narrow spectrum) | 4 |
| HC264 3265 | Beta-lactamase (narrow spectrum) | 4 |
| HC264 3967 | Beta-lactamase (narrow spectrum) | 4 |
| HC264 428 | Beta-lactamase (narrow spectrum) | 4 |
| HC264 53457 | Beta-lactamase (narrow spectrum) | 4 |
| HC264 53502 | Beta-lactamase (narrow spectrum) | 4 |
| HC264 650 | Beta-lactamase (narrow spectrum) | 4 |
| HC55 139 | Quinolone | 4 |
| HC55 1410 | Amikacin/Kanamycin | 4 |
| HC55 2503 | Tetracycline | 4 |
| HC100 119 | Tetracycline | 5 |
| HC167 1681 | Tetracycline | 5 |
| HC167 339 | Tetracycline | 5 |
| HC167 872 | Beta-lactamase (narrow spectrum) | 5 |
| HC19 1571 | Tetracycline | 5 |
| HC19 1764 | Quinolone | 5 |
| HC264 1367 | Beta-lactamase (narrow spectrum) | 5 |
| HC264 147 | Beta-lactamase (narrow spectrum) | 5 |
| HC264 2468 | Tetracycline | 5 |
| HC264 2973 | Streptomycin | 5 |
| HC55 1581 | Tetracycline | 5 |
| HC55 2646 | Beta-lactamase (narrow spectrum) | 5 |
| HC55 918 | Tetracycline | 5 |
| HC100 120 | Tetracycline | 6 |
| HC100 9190 | Streptomycin | 6 |
| HC167 3832 | Amikacin/Kanamycin | 6 |
| HC19 725 | Tetracycline | 6 |
| HC264 105 | Beta-lactamase (narrow spectrum) | 6 |
| HC264 106 | Beta-lactamase (narrow spectrum) | 6 |
| HC264 110 | Beta-lactamase (narrow spectrum) | 6 |
| HC264 1301 | Beta-lactamase (narrow spectrum) | 6 |
| HC264 1691 | Beta-lactamase (narrow spectrum) | 6 |
| HC264 2719 | Beta-lactamase (narrow spectrum) | 6 |
| HC264 2977 | Tetracycline | 6 |
| HC264 883 | Beta-lactamase (narrow spectrum) | 6 |
| HC264 948 | Beta-lactamase (narrow spectrum) | 6 |
| HC55 174 | Quinolone | 6 |
| HC55 220 | Tetracycline | 6 |
| HC55 3297 | Tetracycline | 6 |
| HC55 496 | Tetracycline | 6 |
| HC6 2128 | Tetracycline | 6 |
| HC167 1206 | Tetracycline | 7 |
| HC167 211 | Tetracycline | 7 |
| HC19 278 | Tetracycline | 7 |
| HC264 116 | Beta-lactamase (narrow spectrum) | 7 |
| HC264 27 | Tetracycline | 7 |
| HC264 278 | Beta-lactamase (narrow spectrum) | 7 |
| HC264 322 | Beta-lactamase (narrow spectrum) | 7 |
| HC264 402 | Beta-lactamase (narrow spectrum) | 7 |
| HC264 598 | Beta-lactamase (narrow spectrum) | 7 |
| HC264 798 | Quinolone | 7 |
| HC264 804 | Beta-lactamase (narrow spectrum) | 7 |
| HC264 994 | Beta-lactamase (narrow spectrum) | 7 |
| HC55 215 | Tetracycline | 7 |
| HC55 466 | Tetracycline | 7 |
| HC55 976 | Tetracycline | 7 |
| HC100 1439 | Streptomycin | 8 |
| HC100 406 | Tetracycline | 8 |
| HC100 434 | Streptomycin | 8 |
| HC167 228 | Streptomycin | 8 |
| HC167 5235 | Streptomycin | 8 |
| HC167 815 | Tetracycline | 8 |
| HC264 155 | Beta-lactamase (narrow spectrum) | 8 |
| HC264 2160 | Beta-lactamase (narrow spectrum) | 8 |
| HC264 365 | Quinolone | 8 |
| HC264 426 | Beta-lactamase (narrow spectrum) | 8 |
| HC55 993 | Streptomycin | 8 |
| HC167 1056 | Tetracycline | 9 |
| HC19 2229 | Tetracycline | 9 |
| HC264 140 | Beta-lactamase (narrow spectrum) | 9 |
| HC264 1451 | Beta-lactamase (narrow spectrum) | 9 |
| HC264 2692 | Beta-lactamase (narrow spectrum) | 9 |
| HC264 34 | Beta-lactamase (narrow spectrum) | 9 |
| HC264 349 | Beta-lactamase (narrow spectrum) | 9 |
| HC264 448 | Beta-lactamase (narrow spectrum) | 9 |
| HC264 512 | Beta-lactamase (narrow spectrum) | 9 |
| HC264 6430 | Beta-lactamase (narrow spectrum) | 9 |
| HC264 887 | Beta-lactamase (narrow spectrum) | 9 |
